# Detection of mpox virus in ambient air in a sexual health clinic

**DOI:** 10.1007/s00705-023-05837-z

**Published:** 2023-07-24

**Authors:** Joren Raymenants, Liesbeth Van Gestel, Jasmine Coppens, Tessa De Block, Eugene Bangwen, Jojanneke Rutgers, Matilde Hens, Elise De Vos, Sandra Coppens, Els Keyaerts, Emmanuel André, Antonio Mauro Rezende, Marjan Van Esbroeck, Koen Vercauteren, Laurens Liesenborghs

**Affiliations:** 1 Laboratory of Clinical Microbiology, Department of Microbiology, Immunology and Transplantation, KU Leuven, Herestraat 49, Leuven, 3000 Belgium; 2 Department of Clinical Sciences, Institute of Tropical Medicine, Nationalestraat 155, Antwerp, 2000 Belgium; 3 Clinical Virology Unit, Department of Clinical Sciences, Institute of Tropical Medicine, Antwerp, Belgium; 4 Outbreak Research Team, Department of Clinical Sciences, Institute of Tropical Medicine, Nationalestraat 155, Antwerp, 2000 Belgium; 5 Department of Laboratory Medicine, National reference centre of Respiratory pathogens, University Hospitals Leuven, Herestraat 49, Leuven, 3000 Belgium

**Keywords:** Monkeypox, Saliva, skin, Rectum, Air

## Abstract

Although transmitted mainly through direct (sexual) contact, mpox virus (MPXV) can be detected in ambient air. We explored the use of air sampling for diagnosis or (genomic) surveillance of mpox in a sexual health clinic. For six out of six patients who were infected with MPXV, all four of our ambient air PCR tests were positive. For 14 uninfected patients, PCR was positive in three ambient air samples, albeit with higher cycle threshold (C_t_) values. Genomic sequencing of samples from two positive patients showed matching sequences between air and clinical samples.

**Supplementary Information:**

The online version contains supplementary material available at 10.1007/s00705-023-05837-z.

## Introduction

The 2022 global epidemic of mpox (formerly monkeypox), caused by mpox virus (MPXV), is driven by human-to-human transmission, mainly through close contact during sexual intercourse [1]. Nevertheless, respiratory transmission may also play a role, as the viral load of MPXV is often high in respiratory samples [2, 3]. Indeed, three recent studies demonstrated the presence of MPXV in ambient air. Gould et al. [2] detected MPXV DNA in the vicinity of infected patients in five out of eight samples, using a 50 L/min sampler, but in none of the three samples obtained using a 4 L/min sampler, each for 10 min. Hernaez et al. [3] detected a positive signal in at least one out of three qPCR tests on all 42 ambient air samples collected using a 30 L/min sampler for 30–45 min. In that study, samples were considered positive only if at least two out of three tests were positive with a mean cycle threshold (C_t_) < 35. This resulted in a positivity rate of 64% (27/42). Mellon et al. [4] detected a positive qPCR signal in six out of six qPCR tests on ambient samples taken during visits by one or more mpox-positive patients when using a 200 L/min sampler for 4 hours. In that study, the investigators sampled across several patient visits, did not collect matching clinical samples from all patients, and used a C_t_ value of 40 as the cutoff.

These findings demonstrate not only that MPXV has the potential to become airborne but also that ambient air sampling may be used for surveillance of emerging pathogens such as mpox [5, 6], or even as a non-invasive diagnostic test. While surveillance requires high sensitivity, diagnosis requires additional emphasis on specificity. We therefore further investigated the accuracy of ambient air sampling in mpox diagnosis or surveillance by including negative controls. We also attempted to validate air sampling for genomic surveillance, which would increase the value of this approach.

## Materials and methods

Between September 1 and October 21, 2022, we sampled ambient air in an ambulatory examination room in a sexual health clinic during visits of a total of 20 patients with suspected mpox (Supplementary Fig. S1). During each visit, we sampled air for 20 min using a 200 L/min active air sampler (AerosolSense, Thermo Fisher Scientific), placed at a height of 1.9 m (Supplementary Fig. S2). The HEPA-filter-based ventilation unit generated 10.4 air changes per hour in the examination room. We also collected samples from skin lesions, anal swabs, and saliva. All surfaces in the sampling room were cleaned using alcohol-based rapid disinfectant (Bacillol® AF) between patient visits. Healthcare workers took precautions against contact, droplet, and airborne infection during sampling, using a contact isolation gown, an N95 respirator, a face shield, and nitrile gloves. To test for room contamination, we collected six surface samples in the examination room on one occasion. We collected 19 air samples in an unused room as an additional negative control.

After collection, two individual collection substrates in the sample cartridge and the flocked swabs were suspended separately in 2 ml of ESwab® Amies medium, vortexed at high speed for 10 s, shaken for 20 min, and stored at 4°C. For all samples, 300 µL of the liquefied sample was lysed for 20 min at 56°C using proteinase K, followed by automated DNA extraction (Maxwell®; Promega; 75 µl elution volume). They were then analyzed using two PCR assays: an in-house qPCR assay targeting the MPXV-TNF receptor gene [7, 8] and a commercial MPXV-PCR DM 1.5 kit (Altona Diagnostics, Germany). The air samples collected in an unused room were tested using the in-house PCR assay only.

To confirm that the airborne virus originated from the patient being examined and to evaluate the validity of using air sampling for genomic surveillance, we performed MPXV genome sequencing and single-nucleotide variant (SNV) analysis on matching skin, saliva, and ambient air samples from three selected patients with a high, medium, and low MPXV viral load in ambient air samples (cases 1, 6, and 11, respectively; see Supplementary Table S1 for C_t_ values). Extracted DNA was amplified using primer sets described previously [9], and the resulting amplicons were barcoded using an Oxford Nanopore Ligation Sequencing Kit (SQK-LSK109) with a Native Barcoding Expansion Kit (EXP-NBD104) before sequencing on a MinION flow cell (R9.4.1, Oxford Nanopore Technologies). Sequence read analysis was done using the United States 2022 sequence ON563414.3 as a reference sequence as described previously [7]. Nucleotides in the consensus sequences were considered uncertain (N) if they had a minimum sequencing depth of 5, consisting of individual bases with minimum quality of 7 (Phred score). For the ambient air samples, MPXV reads generated over two independent sequencing runs were assembled. The consensus MPXV genome sequences were aligned with a reference MPXV genome sequence (accession no. ON563414.3) to identify single-nucleotide variants (SNVs).

## Results

Saliva, skin, and anorectal samples from six patients tested positive for MPXV, and these patients were considered infected, while 14 tested negative (Supplementary Table S1). Supplementary Table S2 lists patient characteristics and clinical presentation.

**Fig. 1:**
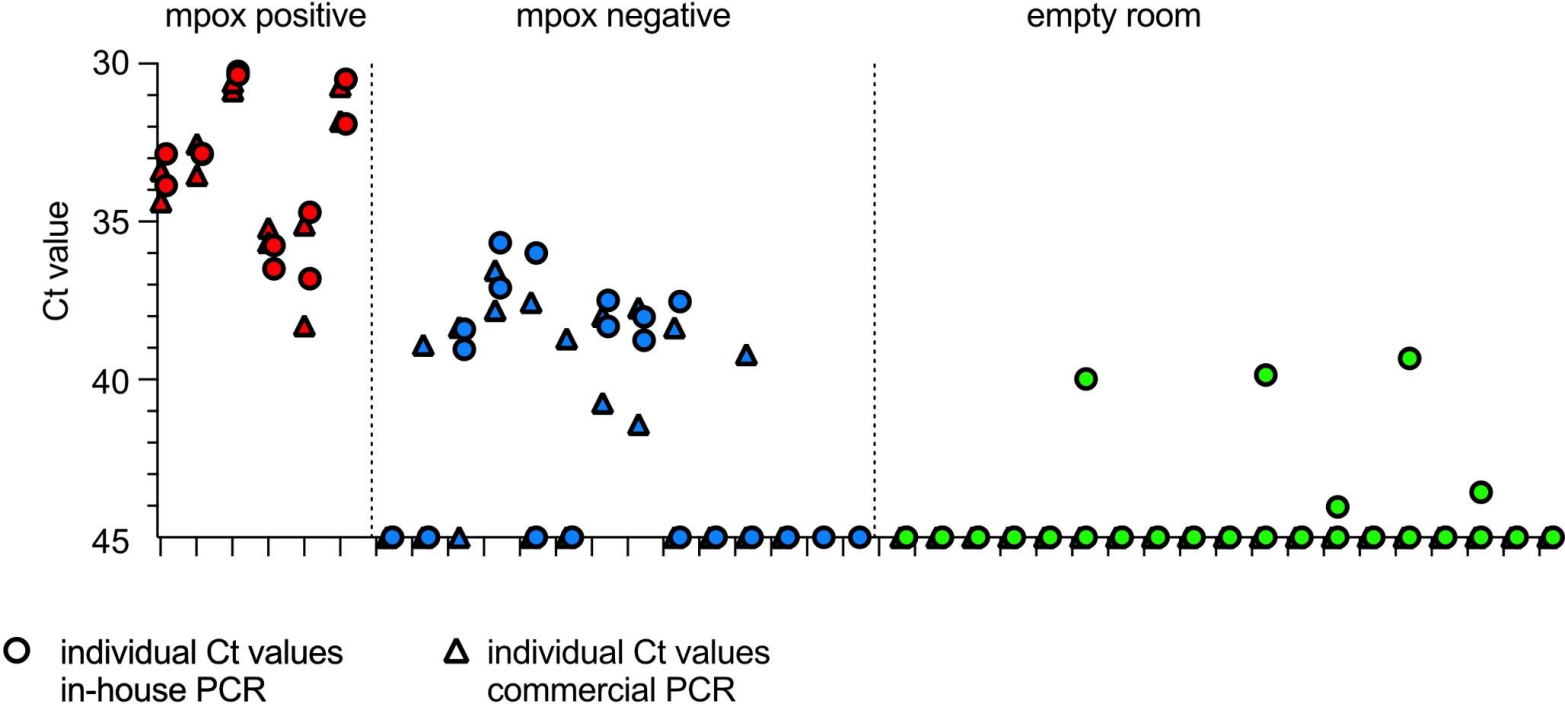
Air sampling results. The figure shows the individual qPCR C_t_ values (*y*-axis) of all ambient air samples (*x*-axis), which were collected either in an outpatient examination room in the presence of infected (red) or uninfected (blue) patients or in an unused room (green). The ticks on the *x*-axis indicate samples taken in the presence of an individual patient. The two collection substrates in each sample cartridge were analyzed separately using both an in-house qPCR assay (D) and an Altona qPCR assay (O). See Supplementary Table S1 for the C_t_ value obtained with each sample, collection substrate, and PCR test.

All PCR assays (24/24) performed on air samples collected in the vicinity of infected patients were positive. Hence, for six out of six cases, all four PCR tests were positive. For air samples collected near uninfected patients, 22 out of 52 PCRs were positive, with air samples from three of 14 uninfected patients testing positive in all four PCR tests. The median C_t_ value in qPCR was 33.14 (IQR 31.58–35.13) for infected patients and 45 (IQR 38.35-45) for uninfected patients. PCR tests performed on 19 ambient air samples taken in an unused room were positive in five out of 76 tests, with C_t_ values between 39.33 and 44.03 (Fig. 1, Supplementary Table S3). None of these negative control samples tested positive in any of the four PCR tests.

Out of six surface samples collected in the examination room, three tested positive in a single PCR test, with C_t_ values ranging from 37.21 to 38.4 (Supplementary Table S4).

**Fig. 2:**
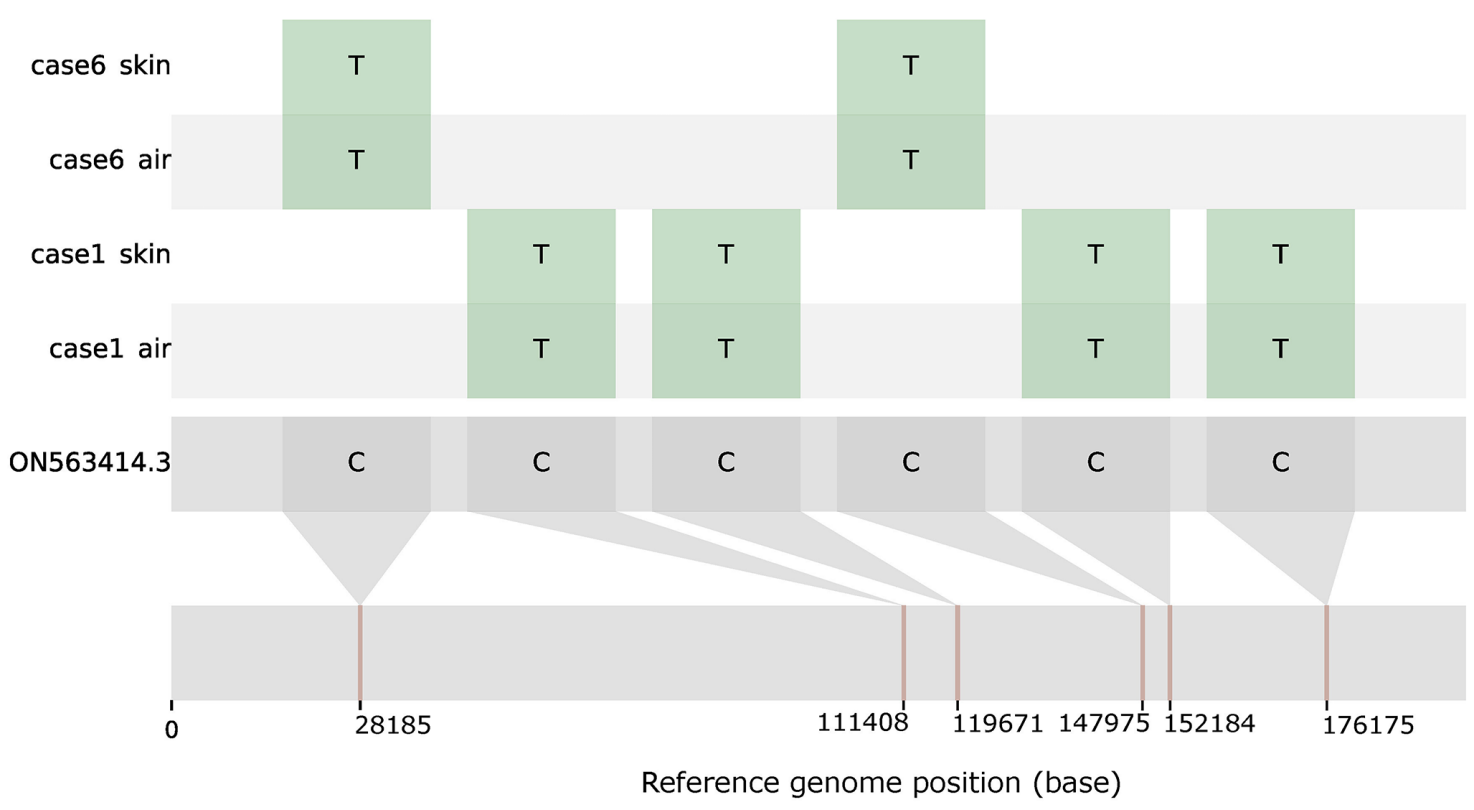
MPXV genome sequencing and SNV analysis. Genome sequences obtained from skin and ambient air samples from cases 1 and 6 matched the United States 2022 sequence (ON563414.3). Bases differing from the reference sequence are shown in green. The positions of genome SNVs relative to the reference genome sequences are indicated at the bottom. The genomic coordinates of the SNVs from skin and ambient air samples and the functional information on each mutation can be found in Supplementary Table S5. The genome sequence alignment file is provided as a separate supplementary file.

Lastly, out of nine independent samples sequenced (one air, one saliva, and one skin sample each from three selected patients), four genome sequences (Fig. 2) that generated less than 10% uncertainly called bases (Ns) were included in the SNV analysis. These included the skin and ambient air samples of cases 1 and 6, which had the lowest ambient air C_t_ values of the three selected cases. The average sequencing depth for these samples was 273.18 (case 1 air, accession no. OQ973326), 1881.90 (case 1 skin, accession no. OQ973327), 1477.93 (case 6 air, accession no. OQ973328), and 1064.54 (case 6 skin, accession no. OQ973329). MPXV genome sequencing and single-nucleotide variant (SNV) analysis demonstrated matching sequences for both sample types (Fig. 2, Supplementary Table S5, Supplementary File “AlignmentFile.aln”).

## Discussion

As did Hernaez et al. [3], we readily detected MPXV DNA in ambient air samples collected in the vicinity of mpox-infected patients during their visit to a sexual health clinic. Using sequencing and SNV analysis, we demonstrated that the MPXV detected in ambient air originated from the visiting patient.

Detection of MPXV DNA in ambient air, however, does not prove aerogenic transmission of the virus. Based on our findings, we can, therefore, not make recommendations regarding precautions against droplet or airborne infections. Indeed, epidemiological data indicate that the 2022 global outbreak was mainly driven by close contact, especially during sexual intercourse [1].

Our findings do indicate, however, that in high-risk settings, ambient air sampling might be used for non-invasive surveillance – including genomic surveillance – or even diagnosis of MPXV. More than for surveillance, however, high test specificity is needed for diagnosis. Due to the waning of the mpox epidemic, we were unable to obtain a sufficient sample size to adequately assess the accuracy of air sampling for diagnostic testing, but we did make some useful observations.

Low sensitivity can be partly overcome by using high-flow samplers and sensitive qPCR platforms like the ones used in this study [5]. Low specificity might result from lab contamination or nonspecific amplification. This may be the reason that we detected mpox virus DNA in five out of 76 air samples collected in an unused room, albeit with high C_t_ values. In addition, false-positive PCRs can result from contamination of a clinical environment by previously evaluated patients. This contamination can result from viral DNA lingering in the air or settling and resuspending. Despite continuous HEPA filtration and surface cleaning between patients, we detected MPXV DNA in 22 out of 52 qPCR tests on air samples obtained near patients without MPXV infection and in three out of six surface swabs [2]. Higher PCR positivity rates and lower C_t_ values in samples collected near infected patients, however, suggest that multiple samplings and setting appropriate C_t_ cutoff values can be used to improve specificity.

## Electronic Supplementary Material

Below is the link to the electronic supplementary material

Supplementary Material 1
