# Supplementary material for: Detection of mpox virus in ambient air in a sexual health clinic
Source: Arch Virol. 2023 Jul 24;168(8):210. doi: 10.1007/s00705-023-05837-z (PMC10366007; doi:10.1007/s00705-023-05837-z)
Supplement: Supplementary file 1 — Supplementary Material 1 [file 705_2023_5837_MOESM1_ESM.docx]

# Supplementary Figures


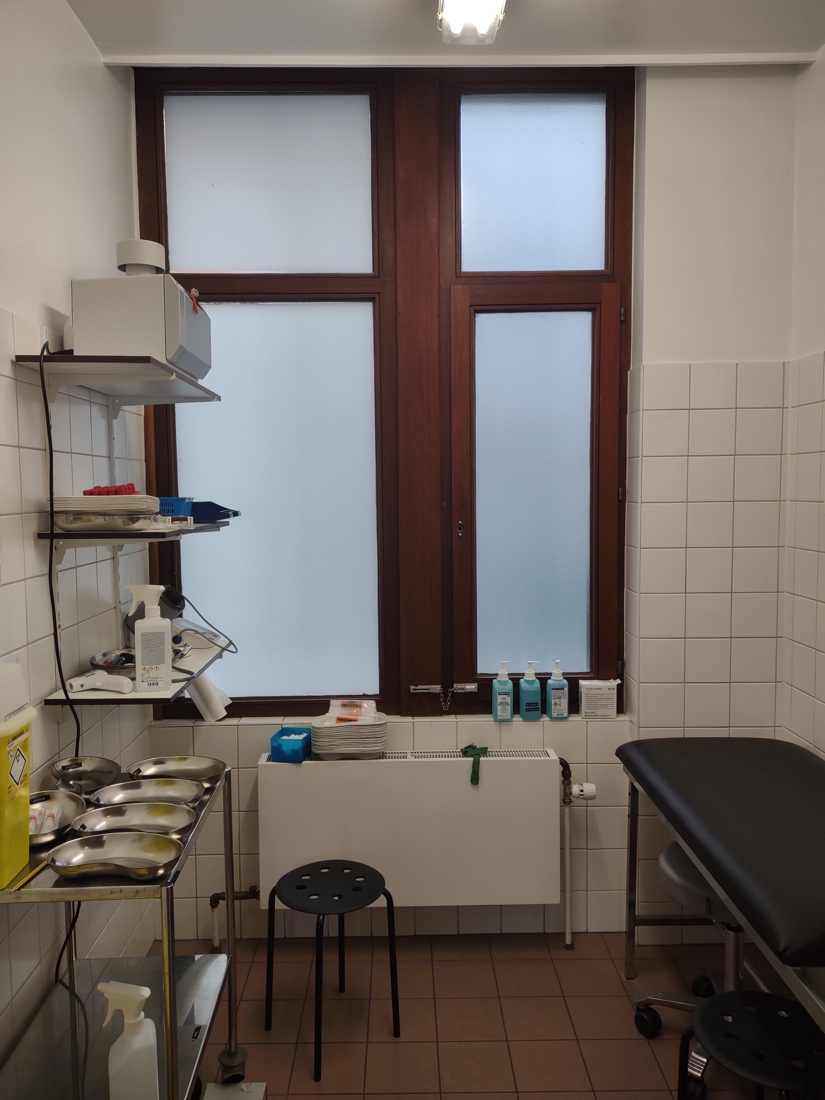


***Supplementary Figure 1. Examination room STI clinic.*** *This figure shows the sampled examination room and the placement of the air sampler. The room is equipped with a HEPA filter, which was last changed two years prior to sampling. The room has a total volume of 12.3m^3^ (2.0*2.15*2.85m). The Heating Ventilation Air Conditioning (HVAC) unit filters 128 m ^3^/hour, which results in 10.4 air changes per hour (ACH). The sampler was placed at a height of 1.9 m, at 2.1 m from the head of the patient examination table. Both the outward window and door to the hallway were kept closed throughout the patient visit.*


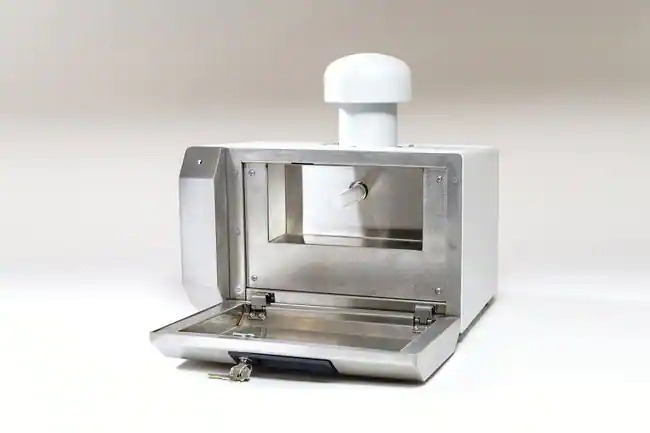

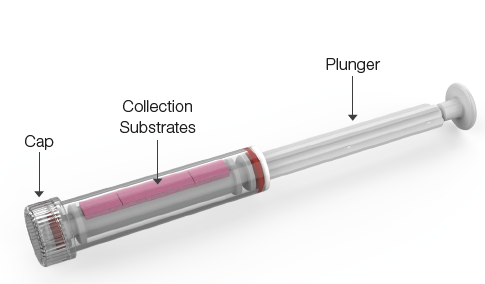


***Supplementary Figure 2. AerosolSense air sampler (Thermo Fisher Scientific®, left) and sample cartridge (Thermo Fisher Scientific®, right).*** *The sampler generates a flow of 200L/min. Sampling time was aimed to be 20min, the duration of a clinical assessment. The sample cartridge contains two individual collection substrates: one on top (shown) and one at the bottom (not shown). These were analysed separately.*

Supplementary Tables

| Mpox diagnosis | Case nr | Lesion | Anal swab | Saliva | Ambient air | | | | |
| --- | --- | --- | --- | --- | --- | --- | --- | --- | --- |
|  |  |  |  |  | Collection substrate 1 | | Collection substrate 2 | | Composite |
|  |  | IH C_t_ | IH C_t_ | IH C_t_ | IH C_t_ | Altona C_t_ | IH C_t_ | Altona C_t_ |  |
| neg | 2 | 45 | 45 | 45 | 45 | 45 | 45 | 45 | neg |
| neg | 3 | 45 | 45 | 45 | 38.91 | 45 | 45 | 45 | neg |
| neg | 5 | 39.05 | 45 | 45 | 38.34 | 38.41 | 45 | 39.05 | neg |
| neg | 7 | 45 | 45 | 45 | 37.81 | 37.09 | 36.55 | 35.67 | **pos** |
| neg | 8 | 45 | 41.75 | 45 | 45 | 45 | 37.56 | 36.00 | neg |
| neg | 10 | 45 | 45 | 45 | 45 | 45 | 38.71 | 45 | neg |
| neg | 13 | 45 | 45 | - | 40.74 | 37.50 | 38.00 | 38.32 | **pos** |
| neg | 14 | 45 | 45 | 45 | 41.42 | 38.76 | 37.73 | 38.02 | **pos** |
| neg | 15 | 45 | 45 | 45 | 45 | 45 | 38.35 | 37.53 | neg |
| neg | 16 | 45 | - | 45 | 45 | 45 | 45 | 45 | neg |
| neg | 17 | 45 | - | 45 | 45 | 45 | 39.21 | 45 | neg |
| neg | 18 | 45 | 45 | - | 45 | 45 | 45 | 45 | neg |
| neg | 19 | 45 | 45 | 45 | 45† | 45 | -† | -† | neg |
| neg | 20 | 45 | 45 | 45 | 45† | 45 | -† | -† | neg |
| **pos** | 1 | 17.02 | 24.17 | 29.80 | 34.37 | 33.86 | 33.40 | 32.85 | **pos** |
| **pos** | 4 | 15.24 | 19.97 | 29.12 | 32.54 | 32.87 | 33.53 | 32.84 | **pos** |
| **pos** | 6 | 16.29 | 17.07 | 25.82 | 30.86 | 30.25 | 30.59 | 30.36 | **pos** |
| **pos** | 9 | 17.60 | 21.75 | 19.74 | 35.20 | 36.50 | 35.66 | 35.76 | **pos** |
| **pos** | 11 | 17.49 | 19.98 | 24.55 | 38.30 | 36.81 | 35.10 | 34.70 | **pos** |
| **pos** | 12 | 19.09 | 18.43 | 21.28 | 30.72 | 30.50 | 31.82 | 31.91 | **pos** |

***Supplementary Table 1.*** *Compilation of MPXV qPCR results from all included mpox suspected patients.* ***‘****IH’ = in-house PCR, ‘Ct’ =’’ PCR cycle threshold, ‘-‘ = not available. qPCR C_t_ values of 45 or above were considered negative and listed as 45. Patients 1,4,6,9 and 11 were considered infected as per clinical protocol, while the remainder was considered not infected. For ambient air samples, the table highlights whether the qPCR was performed on the – randomly numbered – collection substrates 1 or 2. Composite = composite result of air samples. The ambient air composite positive result was defined as detectable MPXV DNA in both used qPCR methods (in house and Altona®) in both collection substrates. All other combinations were scored negative. †individual substrate 1 and 2 PCR data of case 19 and 20 are not available due to pooling of both substrates. Pooled medium PCR data are shown in the table under ‘Collection substrate 1’.*

| Mpox infection | Case nr | Date | Demographics | | Epidemiological risk factors | | | | Symptoms | | | | | Vaccination |
| --- | --- | --- | --- | --- | --- | --- | --- | --- | --- | --- | --- | --- | --- | --- |
|  |  |  | Age | Sex | Recent close contact | MSM | HIV | PREP | Skin | Upper respiratory | Flu like | Rectal | Days since onset |  |
| neg | 3 | 9-1 | 48 | M | - | - | ? | - | + | - | + | - | 7 | - |
| neg | 4 | 9-6 | 37 | M | - | + | + | - | + | - | - | - | 7 | - |
| neg | 7 | 9-7 | 21 | M | + | + | - | - | + | + | + | - | 6 | ? |
| neg | 8 | 9-7 | 44 | M | - | + | - | + | + | - | - | - | 7 | - |
| neg | 9 | 9-8 | 49 | M | + | + | - | + | + | - | - | - | 3 | ? |
| neg | 10 | 9-8 | 34 | M | - | + | - | + | + | + | + | - | 6 | - |
| neg | 11 | 9-20 | 61 | M | - | + | ? | - | + | - | - | - | 7 | + |
| neg | 12 | 9-21 | 57 | M | - | - | ? | - | + | - | - | - | 11 | ? |
| neg | 13 | 9-22 | 33 | F | - | NA | ? | - | + | - | + | - | 6 | - |
| neg | 14 | 9-22 | 36 | M | - | + | - | + | + | - | - | - | 2 | + |
| neg | 16 | 9-26 | 27 | M | ? | + | + | - | + | - | - | - | 6 | + |
| neg | 18 | 10-19 | 19 | F | - | - | - | - | + | - | - | - | 2 | - |
| neg | 19 | 10-21 | 34 | M | ? | + | + | - | + | - | - | + | 9 | - |
| neg | 20 | 10-21 | 49 | M | ? | + | - | + | + | - | - | - | 0 | + |
| pos | 1 | 9-1 | 40 | M | - | + | + | - | + | - | + | + | 7 | - |
| pos | 2 | 9-1 | 26 | M | - | + | - | - | + | + | + | - | 6 | - |
| pos | 5 | 9-6 | 23 | M | - | - | ? | ? | + | + | + | - | 14 | - |
| pos | 6 | 9-6 | 44 | M | - | + | + | - | + | - | + | + | 23 | - |
| pos | 15 | 9-22 | 55 | M | - | + | + | - | + | - | + | + | 5 | + |
| pos | 17 | 9-26 | 44 | M | - | + | + | - | + | + | + | + | 9 | - |

***Supplementary Table 2. Clinical patient characteristics.*** *We included twenty patients with clinical suspicion of mpox from September 1 to October 21, 2022. The waning mpox epidemic hampered recruitment thereafter. ‘+’ = present, ‘-’ = absent, ‘?’ = unknown. Dates are formatted as ‘mo-d’ and relate to 2022. Each patient was subjected to a standard history and clinical examination before sampling. We collected demographical data, including whether patients identified as men who have sex with men (MSM) and whether patients were living with HIV or were using pre-exposure prophylaxis (PREP). We queried whether patients had a recent close contact with a confirmed mpox infected patient. This could be direct skin-contact, sexual contact, the sharing of bedding, and close-range conversation without masks. We assessed the presence, nature and days since onset of symptoms. We defined upper respiratory symptoms as rhinorrhea, nasal congestion, throat ache, painful mouth sores. Flu-like symptom presence required at least one of the following: fever, chills, headache, myalgia, arthralgia. Rectal symptoms implied the presence of rectal pain or symptoms matching with proctitis. We also queried whether they had received a smallpox vaccine at any moment before the assessment.*

| Sample nr | Collection substrate 1 | | Collection substrate 2 | |
| --- | --- | --- | --- | --- |
|  | In-house PCR | Altona PCR | In-house PCR | Altona PCR |
| 1 | nd | nd | nd | Nd |
| 2 | nd | nd | nd | nd |
| 3 | nd | nd | nd | nd |
| 4 | nd | nd | nd | nd |
| 5 | nd | nd | nd | nd |
| 6 | nd | nd | 39.98 | nd |
| 7 | nd | nd | nd | nd |
| 8 | nd | nd | nd | nd |
| 9 | nd | nd | nd | nd |
| 10 | nd | nd | nd | nd |
| 11 | nd | nd | 39.86 | nd |
| 12 | nd | nd | nd | nd |
| 13 | nd | nd | 44.03 | nd |
| 14 | nd | nd | nd | nd |
| 15 | nd | nd | 39.33 | nd |
| 16 | nd | nd | nd | nd |
| 17 | nd | nd | 43.57 | nd |
| 18 | nd | nd | nd | nd |
| 19 | nd | nd | nd | nd |

***Supplementary Table 3. qPCR on air samples in an unused office space.*** *On November 18 and 20 2021, we collected 19 ambient air samples in an unused office space, meaning no patients or employees attended this space in the weeks prior to sampling. We used the same air sampler and duration as in the sampling site. Each of the two collection substrates in the sample cartridge were analyzed using both qPCR types (in-house and Altona). ‘nd’ = not detected. C_t_ values are reported if the qPCR was positive. Five in-house qPCRs generated a positive signal, albeit with high C_t_ values.*

| Sample nr | Surface | In-house PCR |
| --- | --- | --- |
| 1 | Door knob | nd |
| 2 | Light switch | 38.4 |
| 3 | Examination table | 37.6 |
| 4 | Shelf carrying sampler | 37.21 |
| 5 | Screw on sampler to insert air capture medium | nd |
| 6 | Top of air sampler | nd |

***Supplementary Table 4. qPCR on surface samples.*** *‘nd’ = not detected. C_t_ vales are reported if the qPCR was positive. We collected 6 surface samples in the sampling station on November 13 using flocked swabs, which were suspended in 2ml ESwab®. On the in-house qPCR, 2/3 high-touch surfaces (doorknob and examination table) and one possible deposition area (the shelf carrying the sampler) were positive, albeit with high C_t_ values. None were confirmed on the Altona qPCR. Swabs taken from the air sampler tested negative.*

| Reference Nucleotide | Alternative Nucleotide | Genome region | Annotation | Amino acid substitution | Alignment Coordinate | Reference Coordinate (ON563413.3) | Consensus Coordinate: case1 air | Consensus Coordinate: case1 skin | case1 mutation presence | Consensus Coordinate: case6 air | Consensus Coordinate: case6 skin | case6 mutation presence |
| --- | --- | --- | --- | --- | --- | --- | --- | --- | --- | --- | --- | --- |
| C | T | MPXV-USA_2022_MA001-028 | 6R Host immune response repressor (Cop-K7R) VAC B15R-like similar to Vaccinia virus strain Copenhagen K7R | No | 28834 | 28185 | 28342 | 28322 | No | 28325 | 28371 | Yes |
| C | T | MPXV-USA_2022_MA001-113 | 39 kDa immunodominant virion core protein needed for the progression of IV to infectious IMV 39kDa virion core protein (Cop-A4L) A5L similar to Vaccinia virus strain Copenhagen A4L | C171Y | 112057 | 111408 | 111565 | 111545 | Yes | 111548 | 111594 | No |
| C | T | MPXV-USA_2022_MA001-120 | A12R Viral membrane assembly proteins (VMAP) (Cop-A1 1R) similar to Vaccinia virus strain Copenhagen A11R | S18L | 120320 | 119671 | 119828 | 119808 | Yes | 119811 | 119857 | No |
| C | T | MPXV-USA_2022_MA001-153 | A43R Type I membrane glycoprotein (Cop-A43R) membrane glycoprotein similar to Vaccinia virus strain Copenhagen A43R | S180F | 148624 | 147975 | 148123 | 148103 | No | 148106 | 148152 | Yes |
| C | T | Intergenic region | Intergenic region | NA | 152833 | 152184 | 152332 | 152312 | Yes | 152315 | 152361 | No |
| C | T | MPXV-USA_2022_MA001-178 | Ankyrin (Cop-B20R) B17R ankyrin-like | H175Y | 176824 | 176175 | 176315 | 176295 | Yes | 176298 | 176344 | No |

***Supplementary Table 5. Single nucleotide variants (SNVs) in the monkeypox virus genome recovered from skin (*accession: *OQ973327) and ambient air (*accession: *OQ973326) samples of case 1 and skin (*accession: *OQ973329) and ambient air (*accession: *OQ973328) samples of case 6 as compared to reference sequence ON563414.3 (USA, 2022).*** *Out of the 6 SNVs detected, 5 were located in coding regions of the reference genome, and 4 of these resulted in amino acid substitutions. The coding sequence ID and functional annotation were extracted from the reference genome, while the alignment coordinate column can be verified using the supplementary file of the genome alignment. We extracted the coordinates of the sample genomes from the consensus genomes produced for each sample after alignment against the reference.*
